# Fitness but not weight status is associated with projected physical independence in older adults

**DOI:** 10.1007/s11357-016-9911-4

**Published:** 2016-05-05

**Authors:** Luis B. Sardinha, Edilson S. Cyrino, Leandro dos Santos, Ulf Ekelund, Diana A. Santos

**Affiliations:** 1 Exercise and Health Laboratory, CIPER, Faculdade de Motricidade Humana, Universidade de Lisboa, Estrada da Costa, 1499-002 Cruz-Quebrada, Lisboa Portugal; 2 Study and Research Group in Metabolism, Nutrition, and Exercise GEPEMENE, State University of Londrina – UEL, Londrina, Brazil; 3 MRC Epidemiology Unit, University of Cambridge, Cambridge, CB2 0QQ, United Kingdom; 4 Department of Sport Medicine, Norwegian School of Sport Sciences, Oslo, Norway

**Keywords:** Physical fitness, BMI, Waist circumference, Adiposity

## Abstract

Obesity and fitness have been associated with older adults’ physical independence. We aimed to investigate the independent and combined associations of physical fitness and adiposity, assessed by body mass index (BMI) and waist circumference (WC) with the projected ability for physical independence. A total of 3496 non-institutionalized older adults aged 65 and older (1167 male) were included in the analysis. BMI and WC were assessed and categorized according to established criteria. Physical fitness was evaluated with the Senior Fitness Test and individual test results were expressed as Z-scores. Projected ability for physical independence was assessed with the 12-item composite physical function scale. Logistic regression was used to estimate the odds ratio (OR) for being physically dependent. A total of 30.1 % of participants were classified as at risk for losing physical independence at age 90 years. Combined fitness and fatness analysis demonstrated that unfit older adults had increased odds ratio for being physically dependent in all BMI categories (normal: OR = 9.5, 95 %CI = 6.5–13.8; overweight: OR = 6.0, 95 %CI = 4.3–8.3; obese: OR = 6.7, 95 %CI = 4.6–10.0) and all WC categories (normal: OR = 10.4, 95%CI = 6.5–16.8; middle: OR = 6.2, 95 %CI = 4.1–9.3; upper: OR = 7.0, 95 %CI = 4.8–10.0) compared to fit participants that were of normal weight and fit participants with normal WC, respectively. No increased odds ratio was observed for fit participants that had increased BMI or WC. In conclusion, projected physical independence may be enhanced by a normal weight, a normal WC, or an increased physical fitness. Adiposity measures were not associated with physical independence, whereas fitness is independently related to physical independence. Independent of their weight and WC status, unfit older adults are at increased risk for losing physical independence.

## Introduction

With advancing age, physiological changes occur affecting tissues, organ systems, and functions with an impact on physical independence (American College of Sports Medicine et al. 2009), which is defined as having the physical capacity needed to perform common everyday activities without assistance. These activities include simple housework, lifting and carrying objects, negotiating steps, and being able to walk for shopping and errands (Rikli and Jones 2013). With these physiological changes, a long life span brings an increasing risk of loss of independence (Christensen et al. 2008). The functional decline that occurs with aging is a growing problem that affects the health system and the medical treatment of a range of conditions, including musculoskeletal conditions, due to the influence of frailty on mortality, risk of complications and recovery and responsiveness to health interventions (Milte and Crotty 2014).

With an increasing segment of the population getting older, public health efforts should strive for postponing mental and physical disability. Physical fitness is a determinant for preventing or delaying the onset of disability that occurs with aging (den Ouden et al. 2013a; Kuo et al. 2006; Paterson et al. 2004; Rikli and Jones 2013; Sardinha et al. 2015; Wennie Huang et al. 2010). Improved physical fitness components including upper and lower body strength, gait speed, and balance are associated with a lower probability of disability (den Ouden et al. 2011). Further, the benefits of engaging in physical activity programs for preventing disability in the elderly is well documented (den Ouden et al. 2011).

Excessive accumulation of body fat (Davison et al. 2002) and a body mass index (BMI) above 30 kg/m^2^ (Al Snih et al. 2007; Davison et al. 2002; Himes and Reynolds 2012; Reynolds et al. 2005) are associated with an increased likelihood of functional limitations that may influence physical independence, whereas a BMI between 25 and 30 kg/m^2^ may be associated with a lower risk for disability (Al Snih et al. 2007). Although there is evidence that weight reduction interventions can improve physical function among obese older adults (Porter Starr et al. 2014), controversy exists on whether older adults should engage in weight loss programs. Considering data from longitudinal trials examining mortality and body weight, it has been suggested that weight loss interventions in obese older adults should be carefully considered on an individual basis, as it appears maintaining body weight at older age in those who become obese after age 65 years may be beneficial compared with reducing body weight (Bales and Buhr 2008). Recommendations have also been established that weight loss programs in obese older adults (60–79 years old) who experience functional deficits should combine energy intake reduction and exercise to minimize muscle and bone losses (Porter Starr and Bales 2015). Consistent findings have verified that higher fitness levels may counteract the negative effect of obesity mortality risk, particularly in participants with chronic diseases (Barry et al. 2014; Fogelholm 2010; Lavie et al. 2014a; Lavie et al. 2014b; McAuley et al. 2012a; McAuley and Beavers 2014; McAuley and Blair 2011; McAuley et al. 2012b). However, it is unclear whether physical fitness attenuates the impact of obesity on physical independence and vice versa.

Screening for functional status is a preferred preventive approach to avoid physical dependence in older adults (Leipzig et al. 2010). The 12-item composite physical functional scale was developed to identify older adults who are at risk for losing physical independence later in life (Rikli and Jones 1998; Rikli and Jones 2013). The scale discriminates a wide range of functional abilities and has age-adjusted norms that allow for early prediction of independent functioning in later years (90 years old). Projected ability for physical independence represents the projected ability to do a minimum of seven items on the physical functional scale without assistance later in life (defined at >90 years of age) (Rikli and Jones 2013). This approach represents an early diagnostic tool to identify older adults at risk for later functional impairment.

The aim of this investigation was to examine the independent and combined associations of physical fitness and different measures of fatness ((BMI and waist circumference (WC)) on the projected ability for physical independence in Portuguese older adults aged 65 and older. We hypothesized that fit participants will not present an increased risk for physical dependence across weight categories.

## Methods

### Design and subjects

A total of 3496 participants were considered for data analysis (1167 males and 2329 females). Data for the present study were derived from a cross-sectional representative sample of the community-residing Portuguese population aged 65 and older, examined in 2009, including five sampling areas covering the entire mainland of Portugal (Sardinha et al. 2015). The study was carried out in full compliance with the Helsinki Declaration and approved by the local ethics committee. All participants read and signed the consent form before the testing procedures.

### Outcome measures

#### Anthropometry

Participants were weighed to the nearest 0.1 kg and height (Seca, Hamburg, Germany) was measured to the nearest 0.1 cm, according to standardized procedures (Lohman et al. 1988). Body mass index was calculated and classified into normal weight (<25 kg/m^2^), overweight (25–29.9 kg/m^2^), and obesity (≥30 kg/m^2^). Waist circumference was measured with a tape (Seca, Hamburg, Germany) and recorded to the nearest 0.1 cm according to the National Health Examination Survey procedures (CDC 2007). WC was categorized (WHO 2008) as normal WC (males ≤94 cm; females: ≤ 80 cm), middle WC (increased risk: males, 94–102 cm; females: 80–88 cm), and high WC (substantially increased risk: males >102 cm; females: > 88 cm).

#### Physical fitness

Physical fitness was assessed with the *Senior Fitness Test* (Rikli and Jones 2013). Physical fitness parameters included lower and upper body strength, agility/dynamic balance, aerobic endurance, and lower and upper flexibility. The following tests were used to evaluate physical fitness: chair stand (repetitions/30 s), arm curl: women 5 lb. (2.27 kg), men 8 lb. (3.63 kg) (repetitions/30 s); 8-ft up-and-go (s) and the 6­min walk test (m) (6MWT); back scratch (cm) and chair sit-and-reach (cm). Analyses were conducted to verify which physical fitness tests were associated with the projected risk for losing physical independence. The results of each test that was associated with the risk for losing physical independence was standardized (Z-score) by sex, adjusted for age. The sum of all Z-scores was used to compute an overall continuous measure of physical fitness. The continuous variable of physical fitness was then grouped using tertiles and then dichotomized into fit (middle and upper tertile) and unfit (lower tertile), as previously suggested (Lavie et al. 2014b).

#### Physical independence

Having the physical ability needed to live independently was assessed through self-report using the 12-item Composite Physical Function (CPF) scale (Rikli and Jones 1998). The CPF scale describes a wide range of functional abilities, from those associated with basic to instrumental or intermediate to advanced ADL. The scoring requires that participants select one of three responses associated with each of the 12-items: can do (score = 2), can do with help (score = 1), or cannot do (score = 0). Scores are thereafter summed with a potential range between 0 (cannot do any of the 12 tasks) and 24 (can do all 12 tasks independently). The age-adjusted scoring option for defining moderate functioning that reflects projected ability for physical independence at age 90 years, rather than current ability to function independently (Rikli and Jones 2013), was used. Using the age-adjusted scoring, a moderate to high functioning was defined: a: 90+ years: ≥14 (able to perform at least 7 activities without assistance); 80–89 years: ≥16 (able to perform at least 8 activities without help); 70–79 years: ≥18 (able to perform at least 9 activities without help); and 65–69 years: ≥20 (able to perform at least 10 activities without help) (Rikli and Jones 2013). Accordingly, physical independence was dichotomized as low functioning (high risk) and moderate to high functioning (low risk). The use of the age-adjusted option allows for early detection of risk for loss of mobility and independence prior to age 90 years in those who are younger than 90 years (Rikli and Jones 2013).

#### Covariates

Self-reported educational background, medical history, and medication were assessed via interviewer-administered questionnaires. Educational attainment was categorized as follows: (a) no formal education, (b) 4 years of education, (c) 9 years of education, (d) 12 years of education, and (e) higher education. Medical history for hypertension, elevated cholesterol and glycemia, current medication, and the presence of any long-standing condition such as diabetes, asthma, cancer, or cardiac disease and current smoking status were also reported and classified in two categories (yes or no).

### Statistical analysis

Analyses were performed with SPSS (v.22.0, 2013 SPSS Inc., Chicago, Illinois, U.S.A.). Descriptive statistics (mean ± SD) were calculated for all outcome measurements. Independent-sample *t* test or the Kruskal-Wallis test was used to compare means between categories and groups.

Multivariate logistic regression analysis was conducted to analyze the physical fitness variables that were significantly associated with physical independence. Variables that were significant in the model were thereafter used to compute the continuous physical fitness score (Z-score).

Logistic regression analyses, with dichotomized physical independence as the dependent variable, were used to estimate odds ratio (OR) and 95 % confident intervals (CIs) according to exposure categories: fitness, BMI, or WC and combined associations: fitness/BMI or fitness/WC. All analyses were adjusted for age, sex, education, medical history for chronic disease, hypertension, elevated cholesterol or glycemia, and current medication status. Analyses were performed to verify if these covariates were associated with the dependent (physical independence) and the independent variables (fitness, BMI, and WC) (data not shown). Additional models were developed to adjust the analysis for fitness, BMI, or WC, to verify if the associations were independent on the fitness level or the weight and WC status. A variance inflation factor (VIF) for each independent variable (for categorical variables, dummy variables were used) was calculated to evaluate multicollinearity and VIF <5 were accepted (data not shown). The correlation matrix for the logistic regression analyses was examined to ensure that the independent variables were not highly correlated (data not shown). For all tests, significance was set at *p* < 0.05.

## Results

Participants’ characteristics for the all sample and stratified sex and by high and low risk physical independence are summarized in Table 1.

**Table 1:** Participants’ demographic, anthropometric, and physical fitness characteristics according to physical independence group

Variable	All (*n* = 3496)	Low risk (*n* = 3442)	High risk (*n* = 1054)	All females (*n* = 2329)	Low risk females (*n* = 1538)	High risk females (*n* = 791)	All males (*n* = 1167)	Low risk males (*n* = 904)	High risk males (*n* = 263 males)
Age (years)	75.04 ± 7.3	73.5 ± 6.6	78.7 ± 7.8^a^	74.9 ± 7.3	73.1 ± 6.4^b^	78.4 ± 7.6^ab^	75.3 ± 7.4	74.1 ± 6.9	79.6 ± 7.6^a^
*Anthropometry*									
Weight (kg)	69.4 ± 12.2	69.7 ± 11.8	68.7 ± 13.2^a^	66.6 ± 11.5^b^	66.4 ± 10.6^b^	67.0 ± 13.0^ab^	74.9 ± 11.8	75.2 ± 11.5	73.6 ± 12.6
Height (m)	1.57 ± 0.09	1.58 ± 0.1	1.55 ± 0.1	1.53 ± 0.07^b^	1.54 ± 0.07^b^	1.52 ± 0.07^ab^	1.65 ± 0.07	1.66 ± 0.07	1.63 ± 0.08^a^
WC (cm)	95.6 ± 11.6	94.6 ± 11.0	97.9 ± 12.5^a^	94.1 ± 11.8^b^	92.5 ± 10.9^b^	97.4 ± 12.6^ab^	98.5 ± 10.6	98.2 ± 10.1	99.4 ± 12.0
BMI (kg/m^2^)	28.0 ± 4.3	27.8 ± 4.1	28.6 ± 4.9^a^	28.3 ± 4.6 ^b^	28.0 ± 4.3^b^	28.9 ± 5.0^ab^	27.4 ± 3.8	27.4 ± 3.6	27.6 ± 4.3
*Physical fitness*									
6-min walk test (m)	404.4 ± 165.7	459.3 ± 138.2	277.2 ± 153.8^a^	388.9 ± 160.9^b^	447.6 ± 130.4^b^	274.7 ± 153.0^a^	435.4 ± 170.8	479.2 ± 148.5	284.7 ± 156.0^a^
8-ft up-and-go (s)	9.55 ± 7.3	7.3 ± 3.8	14.8 ± 10.1^a^	10.0 ± 7.7^b^	7.4 ± 3.9^b^	15.0 ± 10.4^a^	8.6 ± 6.2	7.0 ± 3.7	14.1 ± 9.2^a^
Chair stand (reps. 30 s^−1^)	13.2 ± 5.8	15.0 ± 4.9	8.9 ± 5.3^a^	13.1 ± 5.8^b^	15.1 ± 5.0	9.1 ± 5.3^a^	13.5 ± 5.6	14.9 ± 4.8	8.6 ± 5.3^a^
Arm curl (reps. 30 s^−1^)	16.0 ± 6.2	17.8 ± 5.4	12.0 ± 5.9^a^	15.7 ± 6.2^b^	17.6 ± 5.4	11.9 ± 5.9^a^	16.7 ± 6.0	18.0 ± 5.4	12.1 ± 5.9^a^
Back scratch (cm)	−17.7 ± 15.5	−14.7 ± 14.0	−24.8 ± 16.5^a^	−15.8 ± 14.9^b^	−12.1 ± 12.9^b^	−23.0 ± 15.8^ab^	−21.6 ± 15.9	−19.2 ± 14.6	−30.0 ± 17.4^a^
Chair sit-and-reach (cm)	−4.8 ± 12.7	−2.5 ± 11.5	−10.1 ± 13.8^a^	−3.2 ± 12.0^b^	−0.3 ± 10.0^b^	−8.8 ± 13.6^ab^	−8.0 ± 13.4	−6.3 ± 12.7	−13.7 ± 14.0^a^
Fitness score	0.000 ± 0.100	0.297 ± 0.799	−0.689 ± 1.074^a^	0.000 ± 1.000	0.331 ± 0.788^b^	−0.643 ± 1.054^ab^	0.000 ± 1.000	0.240 ± 0.816	−0.827 ± 1.124^a^

*WC* waist circumference, *BMI* body mass index, *6MWT* 6 min walking test

^a^Significantly different than low risk group (*p* < 0.05)

^b^Significantly different than males, within the same risk category (*p* < 0.05)

Considering all the samples, 29.8 % of participants were classified as obese (24.3 % of males and 32.6 % females) while 46.0 % were overweight (48.3 % males and 44.9 % females). In addition, 23.7 % (30.8 % of males and 20.2 % of females) were classified in the middle WC category while 59.0 % (36.4 % of males and 70.2 % of females) were in the upper WC category. Older adults categorized as high risk for losing physical independence had higher BMI and WC and inferior results in the physical fitness tests when compared to the low risk group (Table 1).

Logistic regression analyses were performed to identify the fitness test variables that were significantly associated with physical independence. Table 2 shows the odds ratio for each test that was associated with high risk for losing physical independence. The model was adjusted for age, sex, education, medical history for chronic disease, hypertension, elevated cholesterol, elevated glycemia, smoking status, and current medication status.

**Table 2:** Logistic regression model to determine the associations between physical fitness tests with high risk for losing physical independence

	Model a	Model b^a^
	OR (95 % CI)	OR (95 % CI)
Chair Stand (reps/30 s)	0.930 (0.904–0.955)*	0.928 (0.903–0.954)*
Arm curl (reps/30 s)	0.956 (0.936–0.977)*	0.956 (0.936–0.977)*
6­minute walk test (m)	0.996 (0.996–0.997)*	0.996 (0.996–0.997)*
8-ft up-&-go (s)	1.090 (1.067–1.112)*	1.091 (1.069–1.114)*
Chair sit-&-reach (cm)	0.990 (0.982–0.999)*	0.990 (0.981–0.998)*
Back Scratch (cm)	0.996 (0.989–1.003)	not included

All models were adjusted for age, sex, education, medical history for chronic disease, hypertension, elevated cholesterol or glycemia, smoking status, and current medication status.

*< 0.05

^a^Model b was developed using only significant fitness test predictors

Each repetition completed in the chair stand test (*p* < 0.001) and in the arm curl (*p* < 0.001) test were associated with a 7.2 and a 4.4 % lower odds of being at high risk for losing physical independence. Each meter walked in the 6MWT (*p* < 0.001) was associated with a 0.4 % reduction and each centimeter (*p* = 0.012) in the chair sit-and-reach with a 1.0 % reduction in the odds of being at high risk for losing physical independence. Similarly, for each second increase in the 8-ft up-and-go test, there was a 9.1 % increase in the odds of being at risk for losing physical independence (*p* < 0.001).

Logistic regression analysis demonstrated that, with the exception of the back scratch tests (*p* = 0. 225), all physical fitness tests were significantly associated (*p* < 0.05) with high risk for losing physical independence. Accordingly, a continuous fitness score was created using the age-adjusted standardized values of the chair stand, the arm curl, the 6MWT, the 8-ft. up-and-go, and the chair sit-and-reach test, by sex.

**Fig. 1:**
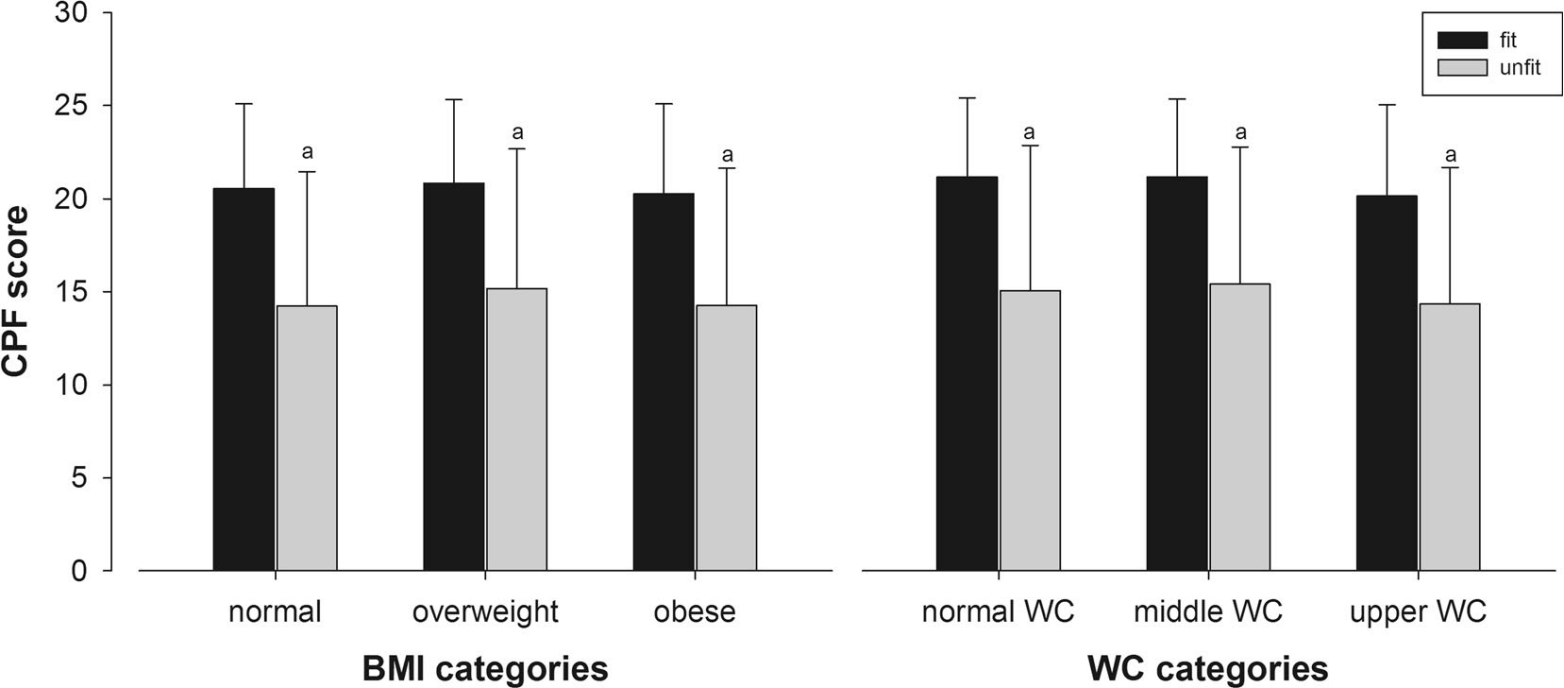
Results for the Composite Physical Function (CPF) scale stratified by physical fitness and body ass index category (left panel) and by physical fitness and waist circumference (*right panel*). Abbreviations: *CPF*, composite physical function scale; *BMI*, body mass index; WC, waist circumference. *Lowercase letter a* means significant differences with fit/normal weight, with fit/overweight, and with fit/obese. Results are presented as mean and standard error

Figure 1 shows the results for the CPF scale stratified by fitness and BMI or WC category.

Higher scores in the CPF scale (*p* < 0.001) were found in fit participants in all BMI and WC categories when compared to all participants that were categorized as unfit (lower tertile).

Odds ratios for being at projected risk for losing physical independence according to BMI, WC, and fitness categories are shown in Table 3.

**Table 3:** Odds ratios for being at risk of losing physical independence

	Number of subjects	*N* (%) at risk	Model 1	Model 2	Model 3	Model 4	Model 5
*Fitness*				*a*	*a*, *b*	*a*, *c*	*a*, *b*, *c*
Upper	1165	657 (56.4)	1.00 (reference)	1.00 (reference)	1.00 (reference)	1.00 (reference)	1.00 (reference)
Middle	1166	252 (21.6)	1.94 (1.55–2.42)	2.77 (2.17–3.55)*	2.75 (2.15–3.52)*	2.72 (2.13–3.48)*	2.72 (2.13–3.48)*
Low	1165	145 (12.4)	9.10 (7.38–11.21)*	13.01 (10.25–16.53)*	12.71 (10.01–16.15)*	12.34 (9.70–15.69)*	12.37 (9.72–15.74)*
*BMI*				*a*	*a*, *c*	*a*, *d*	*a*, *c*, *d*
< 25.0 kg/m2	844	244 (28.9)	1.00 (reference)	1.00 (reference)	1.00 (reference)	1.00 (reference)	1.00 (reference)
25.0–29.9 kg/m2	1609	430 (26.7)	0.90 (0.75–1.08)	0.97 (0.79–1.19)	0.74 (0.60–0.93)*	0.91 (0.72–1.15)	0.84 (0.65–1.08)
> 29.9 kg/m2	1043	380 (36.4)	1.41 (1.16–1.71)*	1.47 (1.18–1.83)*	0.84 (0.63–1.12)	1.15 (0.89–1.48)	0.97 (0.70–1.34)
*WC*				*a*	*a*, *b*	*a*, *d*	*a*, *b*, *d*
Low	605	134 (22.1)	1.00 (reference)	1.00 (reference)	1.00 (reference)	1.00 (reference)	1.00 (reference)
Middle	830	191 (23.0)	1.06 (0.82–1.35)	0.99 (0.75–1.30)	0.92 (0.70–1.21)	0.78 (0.57–1.07)	0.73 (0.53–1.00)
Upper	2061	729 (35.4)	1.92 (1.56–2.38)	1.54 (1.21–1.96)*	1.26 (0.95–1.67)	0.96 (0.73–1.26)	0.81 (0.59–1.11)

a = Model adjusted for age, sex, education, medical history for chronic disease, hypertension, elevated cholesterol or glycemia, smoking status, and current medication status; b = model adjusted for body mass index (as continuous variable); c = model adjusted for waist circumference (as continuous variable); d = model adjusted for fitness (as continuous variable)

*BMI* body mass index, *WC* waist circumference

*< 0.05

After adjustment for age, sex, education, medical history for chronic disease, hypertension, elevated cholesterol or glycemia, smoking status, and current medication status (model 1), participants who were obese (OR = 1.47, *p* = 0.001) or in the upper WC category (OR = 1.54, *p* < 0.001) were at projected risk for losing physical independence while those categorized as overweight (OR = 0.97, *p* = 0.780) and in the middle WC category (OR = 0.99, *p* = 0.962) had no significantly increased risk compared with the reference group (normal BMI and normal WC, respectively).

Following adjustment for physical fitness, neither BMI nor WC categories (*p* > 0.05) were associated with increased odds of being at projected risk for losing physical independence. In contrast, belonging to the middle (OR = 2.72, *p* < 0.001) and lower fitness tertiles (OR = 12.37, *p* < 0.001) was associated with significantly increased odds for being at projected risk for losing physical independence, compared to the highest fitness tertile, and this association was independent of both BMI and WC.

**Fig. 2:**
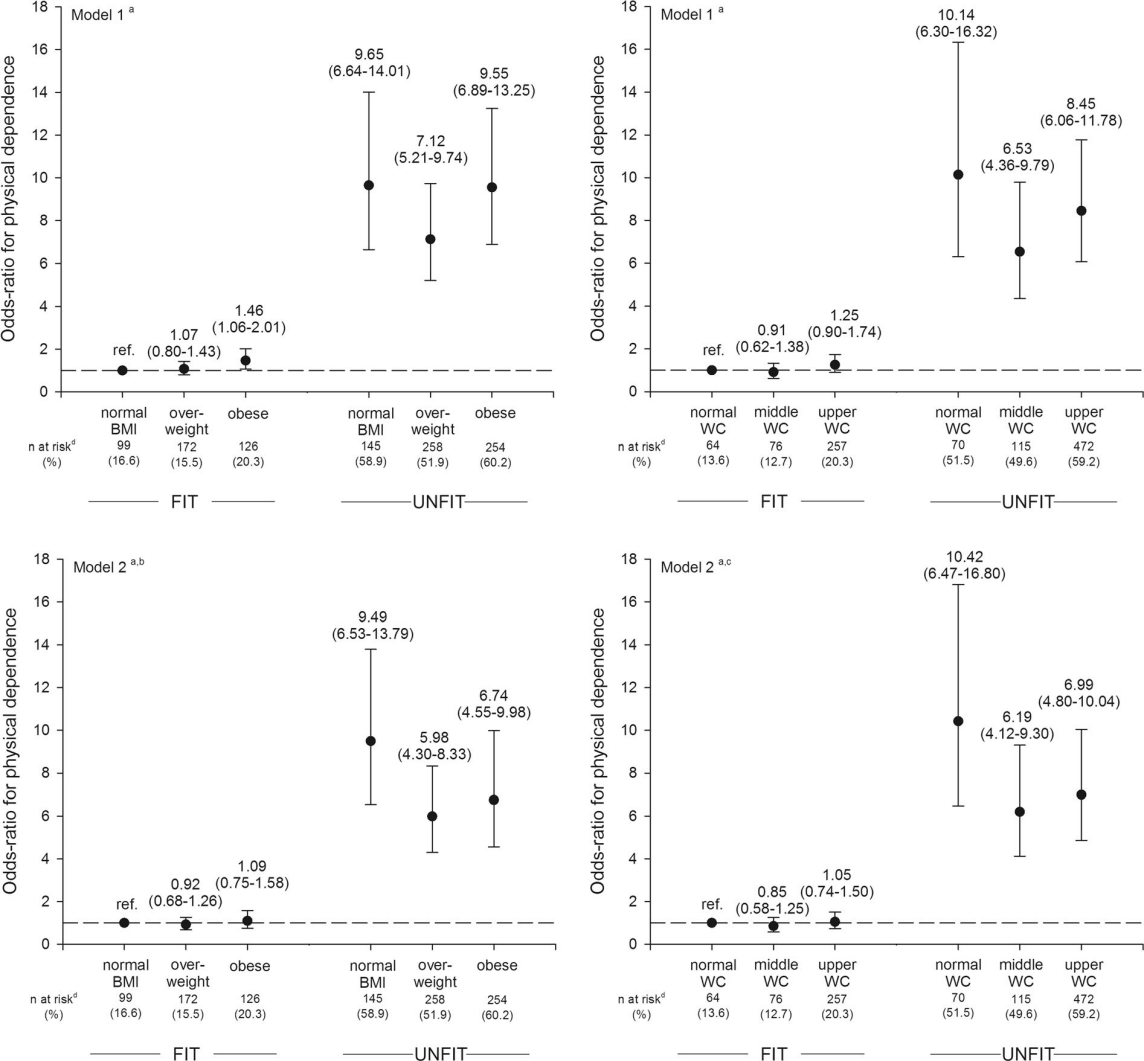
Combined effects of fitness and body mass index (BMI) (*left panel*) and waist circumference (right panel), on the odds of being at risk for losing physical independence. Abbreviations: *BMI*, body mass index; *WC*, waist circumference. *a =* model adjusted for age, sex, education, medical history for chronic disease, hypertension, elevated cholesterol or glycemia, and current medication status; *b =* model adjusted for body mass index (as continuous variable); *c* = model adjusted for waist circumference (as continuous variable); *d* = number of participants at risk for physical dependence

The combined associations of fitness and adiposity measures (BMI and WC) with the risk for losing physical independence are illustrated in Fig. 2.

In combined association analyses, those who were categorized as unfit, regardless of their weight status, had substantially increased odds for being categorized as projected risk of losing physical independence compared with the reference group (high fit participants with normal BMI). Interestingly, fit participants that were overweight or obese did not substantially increase the risk compared with the reference group.

A similar pattern was observed when analyzing the combined effects of fitness and WC on physical independence. In fit participants, the OR for being at risk for losing physical independence did not differ across WC groups. In unfit participants, significantly increased OR was observed for unfit with normal WC (OR = 10.42, *p* < 0.001), for unfit with middle WC (OR = 6.19, *p* < 0.001), and for unfit with high WC (OR = 6.99, *p* < 0.001) compared with fit and normal-weight participants.

Additional analyses were conducted using the continuous standardized variables of fitness, BMI, and WC as the independent variables (Table 4).

**Table 4:** Independent and combined logistic regression analysis for the associations between continuous variables of fitness, body mass index, and obesity with being at risk of losing physical independence

	β (SE)	OR (95 % CI)	Β (SE)^a^	OR (95 % CI)^a^
*Independent Models*				
zFitness	−1.186 (0.051)*	0.31 (0.28–0.34)*	−1.321 (0.057)*	0.27 (0.24–0.30)*
zBMI	0.213 (0.037)*	1.24 (1.15–1.33)*	0.194 (0.041)*	1.21 (1.12–1.32)*
zWC	0.292 (0.038)*	1.34 (1.24–1.44)*	0.286 (0.041)*	1.33 (1.23–1.44)*
*Combined Fitness and BMI Model*				
zFitness	−1.176 (0.051)*	0.31 (0.28–0.34)*	−1.312 (0.057)*	0.27 (0.24–0.30)*
zBMI	0.117 (0.041)*	1.12 (1.04–1.22)*	0.092 (0.046)*	1.10 (1.00–1.20)*
*Combined Fitness and WC Model*				
zFitness	−1.166 (0.051)*	0.32 (0.28–0.35)*	−1.304 (0.057)*	0.27 (0.24–0.30)*
zWC	0.115 (0.042)*	1.12 (1.03–1.22)*	0.091 (0.047)	1.10 (0.99–1.20)

*BMI* body mass index, *WC* waist circumference

*< 0.05

^a^Coefficients presented are adjusted for age, sex, education, medical history for chronic disease, hypertension, elevated cholesterol or glycemia, smoking status, and current medication status

The independent analysis demonstrated that for each standard deviation increase in fitness, a 69 % decrease in the risk for losing physical independence was observed. The results for BMI and WC were less pronounced. For each standard deviation increase, the risk for losing physical independence increased by 24 and 34 %, respectively. In combined analysis, it was observed that fitness remained significantly associated with the risk for physical dependence after adjusting for BMI or for WC. Fatness analysis with BMI and WC demonstrated that when adjusting for current fitness levels, only BMI remained significantly associated with projected physical independence.

## Discussion

The current study examined the independent and combined associations of BMI, WC, and physical fitness on projected ability for physical independence. Combined associations between physical fitness, BMI, and WC on physical independence indicated that the risk for losing physical independence is dependent on physical fitness rather than overall and abdominal adiposity status. Accordingly, our results suggest that fit individuals, who are overweight or obese or had an increased WC, did not present an increased projected risk for physical independence. Yet, for all BMI and WC categories, older adults that were unfit were at higher risk of losing physical independence.

Some investigations suggesting that obesity is associated with impaired functional status have not considered physical fitness in their analysis (Davison et al. 2002; Himes and Reynolds 2012; Reynolds et al. 2005). Regardless, evidence from cross-sectional (Kuo et al. 2006; Rikli and Jones 2013; Sardinha et al. 2015) and longitudinal (den Ouden et al. 2013a; Paterson et al. 2004; Wennie Huang et al. 2010) data verified that physical fitness is associated with functional status. Kuo et al. (2006) have identified that fitness parameters including peak leg power and habitual gait speed were associated with varying domains of late-life disability. Other authors have proposed standards for selected fitness parameters that were associated with physical independence (Rikli and Jones 2013; Sardinha et al. 2015).

One previous study with a 10-year follow-up period (den Ouden et al. 2013b) identified several factors that predicted disability later in life. The final prediction model included muscle strength (isometric handgrip and leg extension), the number of chronic diseases, age, gender, and socioeconomic status, but other candidate predictors, including BMI and other functional fitness parameters (standing balance, 8-ft. walk and ability to rise from a chair), were not significant in the final model. Similarly an 8-year follow-up study (Paterson et al. 2004) identified that, in participants aged 55 to 86 years old, cardiorespiratory fitness predicted physical independence, but neither BMI nor other functional fitness parameters (strength, flexibility, walking pace) were related to increased odds of becoming dependent. These previous studies (den Ouden et al. 2013b; Paterson et al. 2004) emphasize that a multifactorial approach is needed to accurately predict physical independence. However, overall adiposity assessed as BMI appears not to be a significant predictor of physical independence when fitness variables are taken into account. Interestingly, there seems to be no consensus on whether cardiorespiratory fitness, muscle strength, or functional fitness is the most important determinant of physical independence. In the current investigation, all fitness variables except for upper body flexibility were associated with projected physical independence. In older participants, it may be more important to assess physiologic parameters that support physical mobility (Kuo et al. 2006; Rikli and Jones 1999; Rikli and Jones 2013; Sardinha et al. 2015). Accordingly, in the present study, physical fitness was considered as a composite variable that includes selected functional fitness parameters that are associated with physical independence.

The independent and combined associations between fitness and fatness have been demonstrated for other health outcomes with fit (higher cardiorespiratory fitness) patients with hypertension, the metabolic syndrome, and type 2 diabetes (Lavie et al. 2015) and cardiovascular diseases (Barry et al. 2014; Lavie et al. 2015; Lee et al. 2011) presenting a better clinical prognosis and a lower mortality risk when compared to unfit participants, regardless of their BMI status. Our results extend these previous observations and suggest that high fitness rather than fatness are important for physical independence in older adults. For a given BMI, physically fit persons may have lower adiposity and perhaps one of the mechanisms by which physical fitness reduces health risks that are associated with BMI is by decreasing fat-to-lean mass ratio and also by decreasing visceral-to-subcutaneous fat ratio (Fogelholm 2010).

Exercise interventions in older adults, even without weight loss, can lead to benefits in physical fitness and functional status, while preserving bone mineral density and lean body mass (Villareal et al. 2011). Compared to combined diet and exercise interventions, similar benefits in functional status can be obtained with an exercise intervention alone (Villareal et al. 2011). In older adults, lifestyle weight loss interventions, combining diet and exercise, can lead to positive changes in physical function, in spite of the lean body mass and bone mineral losses, although the clinical significance of these changes is unclear (Waters et al. 2013). On the other hand, there is still some controversy whether or not weight loss should be prescribed in later life. Weight loss interventions are not recommended for all older population; decisions about whether or not a weight loss intervention should be instituted for obese older adults should be carefully considered on an individualized basis, with special attention to the weight history and the medical conditions of each person (Bales and Buhr 2008).

Our findings are promising for all older adults, including those who are not recommended to lose weight, as all can experience benefits in their physical independence status by increasing their fitness level through increases in everyday physical activity. Physical activity guidelines advocate that all older adults should engage in at least 150 min/week of moderate-intensity aerobic physical activity and that muscle-strengthening activities, involving major muscle groups, should be done on 2 or more days a week. In older adults, there may be some safety issues regarding physical activity intensity. Exercise-associated musculoskeletal injuries may occur in physical activity interventions (Villareal et al. 2011). Regardless, we have previously demonstrated that benefits in physical fitness (Sardinha et al. 2014) and abdominal obesity (Júdice et al. 2015) can be attained by breaking up sedentary time more often, independently of the time spent in physical activity at a moderate intensity. Accordingly, when older adults cannot do the recommended amounts of physical activity due to health conditions, they may achieve health benefits by reducing the time spent in sedentary behaviors and thereby increase their overall amount of physical activity.

Strength of the study includes a large nationally representative sample of Portuguese older adults which increases the generalizability of our results. However, the study also presents some limitations. The cross-sectional design prohibits us from determining causality and direction of association. The use of BMI as a measure of overall adiposity may be a limitation given that there is a progressive increase in the fat to lean ratio by increasing age (Prentice and Jebb 2001). Accordingly, the associations observed in the current study may not fully reflect the association between fatness and physical independence, as differences in the fat to lean ratio for the same BMI may occur. Further, we cannot exclude the possibility that our results are explained by poorly measured or unmeasured confounders. For example, educational attainment was used as a proxy of socioeconomic status.

In conclusion, projected ability for physical independence later in life (90 years old) may be enhanced by a normal weight, a normal WC, and especially by high levels of physical fitness. Unfit older adults are at increased risk for losing physical independence regardless of their overall and abdominal obesity status. Practitioners should attempt to encourage older participants to adhere to exercise programs that are designed to enhance physical fitness, rather than focusing on reducing body weight, as benefits in physical independence are likely achieved, independent of weight status.
